# Fatty Acid Profile and Antioxidant Status Fingerprint in Sarcopenic Elderly Patients: Role of Diet and Exercise

**DOI:** 10.3390/nu11112569

**Published:** 2019-10-24

**Authors:** Paola Antonia Corsetto, Gigliola Montorfano, Catherine Klersy, Luca Massimino, Vittoria Infantino, Giancarlo Iannello, Milena Anna Faliva, Henry Lukaski, Simone Perna, Tariq A. Alalwan, Angela Maria Rizzo, Mariangela Rondanelli

**Affiliations:** 1 Department of Pharmacological and Biomolecular Sciences, Università degli Studi di Milano, 20133 Milano, Italy; paola.corsetto@unimi.it (P.A.C.); gigliola.montorfano@unimi.it (G.M.); 2 Biometry and Clinical Epidemiology Service, Fondazione IRCCS Policlinico San Matteo, 27100 Pavia, Italy; klersy@smatteo.pv.it; 3 Division of Neuroscience, San Raffaele Scientific Institute, 20132 Segrate, Italy; luca.massimino@hsr.it; 4 Clinical Nutrition and Endocrine Unit, Azienda di Servizi alla Persona di Pavia, University of Pavia, 27100 Pavia, Italy; viriainfantino@hotmail.it (V.I.); milena.faliva@gmail.com (M.A.F.); 5 General Management, Azienda di Servizi alla Persona di Pavia, University of Pavia, 27100 Pavia, Italy; direttoregenerale@asppavia.it; 6 Department of Kinesiology and Public Health Education, Hyslop Sports Center, University of North Dakota, Grand Forks, ND 58202, USA; 7 Department of Biology, College of Science, University of Bahrain, Sakhir Campus P.O. Box 32038, Kingdom of Bahrain; sperna@uob.edu.bh (S.P.); talalwan@uob.edu.bh (T.A.A.); 8 IRCCS Mondino Foundation, 27100 Pavia, Italy; 9 Department of Public Health, Experimental and Forensic Medicine, University of Pavia, 27100 Pavia, Italy

**Keywords:** fatty acid, antioxidants, elderly, sarcopenia, frailty, exercise, supplement

## Abstract

Plasma fatty acids (FAs) and oxidant status contribute to the etiology of sarcopenia in the elderly concurring to age-related muscle loss and elderly frailty through several mechanisms including changes in FA composition within the sarcolemma, promotion of chronic low-grade inflammation, and insulin resistance. The aim of this study was to determine the FA profile and pro-antioxidant status in sarcopenic frail elderly patients enrolled in a nutritional and physical activity program and to evaluate their correlation with clinical markers. Moreover, the possible changes, produced after a short-term clinical protocol, were evaluated. Plasma and erythrocyte FA composition and pro-antioxidant status were analyzed in sarcopenic elderly subjects recruited for the randomized clinical study and treated with a placebo or dietary supplement, a personalized diet, and standardized physical activity. Subjects were tested before and after 30 days of treatment. Pearson correlations between biochemical parameters and patients’ characteristics at recruitment indicate interesting features of sarcopenic status such as negative correlation among the plasma FA profile, age, and physical characteristics. Physical activity and dietetic program alone for 30 days induced a decrease of saturated FA concentration with a significant increase of dihomo-gamma-linolenic acid. Supplementation plus physical activity induced a significant decrease of linoleic acid, omega-6 polyunsaturated FAs, and an increase of stearic and oleic acid concentration. Moreover, glutathione reductase activity, which is an indicator of antioxidant status, significantly increased in erythrocytes. Changes over time between groups indicate significant differences for saturated FAs, which suggest that the amino acid supplementation restores FA levels that are consumed during physical activity. A relationship between FA and clinical/metabolic status revealed unique correlations and a specific metabolic and lipidomic fingerprint in sarcopenic elderly. The results indicate the positive beneficial role of supplementation and physical activity on plasma FA status and the antioxidant system as a co-adjuvant approach in sarcopenic, frail, elderly patients.

## 1. Introduction

Sarcopenia is a multifactorial syndrome emerging from changes in muscle morphology, oxidative stress, inflammation, physical activity, and diet [1,2]. Oxidative stress moderates aging and exacerbates age-associated diseases [3,4,5]. It is an etiological factor in the development of sarcopenia [6]. Elevated metabolic activity of skeletal muscle increases the production of reactive oxygen species (ROS), which increases oxidative stress [7]. Antioxidant enzymes, therefore, are important for skeletal muscle adaptation to increased physical activity with contrasting experimental findings revealing both a compensatory induction of antioxidant activity [8] as well as antioxidant systems’ dysfunction [9] during aging. Superoxide dismutase (SOD) is responsible for the dismutation of the superoxide anion, O_2_^.^, and the elimination of cellular oxidative stress. The absence of the cytosolic SOD1 isoform significantly decreases the lifespan [10]. Recent findings reveal that high levels of oxidative stress accelerated sarcopenia due to O_2_^.^ induced neuromuscular degeneration and mitochondrial dysfunction in SOD1-deficient mice [4,11,12]. Pansarasa et al. [8] proposed an association between ROS activity and age-related changes in human skeletal muscle, while Mecocci et al. [13] demonstrated age-related oxidative damage and significant loss of muscle mass. Increased levels of hydrogen peroxide (H_2_O_2_) have been found in aging skeletal muscle. Capel et al. [14] demonstrated an increase in mitochondrial H_2_O_2_ release in the tibialis anterior (TA) muscles of 24-month old male Wister rats with sarcopenia when compared to 4.5-month controls. Capel et al. [15] confirmed increased mitochondrial H_2_O_2_ release in the vastus lateralis muscle of elderly subjects. Animal models have been critical for our understanding of human sarcopenia [16]. Mitochondria obtained from aged rodent muscle fibers displayed several functional abnormalities by explaining the increased proteolysis associated with ROS overproduction and vulnerability to apoptosis exhibited by the sarcopenic muscle.

Noteworthy, these deleterious changes appear to be related to modifications of the fatty acid (FA) profile of mitochondrial phospholipids [17]. Aging was found to be correlated with a decrease of stearic acid, which leads to a reduction in the percentage of saturated FAs. On the other hand, it raised the proportion of oleic acid and total monounsaturated FAs. The total content of polyunsaturated FAs was not modified, but the nature of these FAs was changed.

Dietary fats are a major source of energy for resting and working muscle and can exert effects on age-related muscle loss through several mechanisms. These include changes in FA composition within the sarcolemma (muscle cell membrane), promotion of chronic low-grade inflammation, and insulin resistance [18,19,20].

Lipina and Hundal [21] in their review discussed the role of FAs and their derived lipid intermediates in the regulation of skeletal muscle mass and function, which underlies the importance of lipidomic evaluations related to the possible lipid influence on muscle metabolism, structure, and strength. Despite the heightened awareness of the role of oxidative status in the development of sarcopenia, as well as fat function in muscle energy production during exercise and the potential for fats to influence mechanisms associated with muscle loss, there is a paucity of data integrating these factors in human sarcopenia.

The aim of this study was to determine the FA profile and pro-antioxidant status in sarcopenic, frail, elderly patients enrolled in a nutritional and physical activity program in order to evaluate their correlation with clinical markers. Moreover, this study has evaluated the possible changes produced after a short-term clinical supplementation protocol designed to counteract elderly sarcopenia, promoting muscle accretion compared to placebo.

Relationship between FA and clinical/metabolic status, determined by physical and hemato-chemical parameters, body composition assessed with bioimpedance, and psychological assessment, revealed interesting correlations and a specific metabolic and lipidomic fingerprint in sarcopenic elderly. The results indicate the positive beneficial role of supplementation and physical activity on the plasma FA status and antioxidant system as a co-adjuvant approach in sarcopenic, frail, elderly patients.

## 2. Materials and Methods

### 2.1. Participants

The Ethics Committee of the Department of Internal Medicine and Medical Therapy and the Institutional Review Board at the University of Pavia approved this study. All participants were informed about the study, gave their written consent, and were guaranteed confidentiality and anonymity (ClinicalTrials.gov Identifier: NCT02402608). 

Subjects were elderly men and women admitted to the geriatric physical medicine and rehabilitation division at the Istituto Santa Margherita, Azienda di Servizi alla Persona di Pavia (Pavia, Italy).

All participants underwent a medical examination before enrolment in the study. Eligible patients were 65 years or older with appendicular skeletal fat-free mass (FFM) divided by height squared two standard deviations (SD) below the mean of young adults [22] or relative muscle mass <7.26 kg/m^2^ for men and <5.5 kg/m^2^ for women. Patients were not affected by acute illness, severe liver, heart or kidney dysfunction, and had a stable body weight over the preceding six months. Subjects with altered glycometabolic control, dysthyroidism, other endocrinopathies, and cancers, as well as patients treated with steroids and heparin, or with total walking incapacity were excluded. At enrollment, the functional status was assessed using instrumental activities of daily living (IADLs), and cognitive status was assessed with the Mini-Mental State Examination (MMSE) [23].

The patients’ fasting blood biochemical indices of nutritional and health status (total and low density lipoprotein (LDL) serum cholesterol, triglycerides, high density lipoprotein (HDL) cholesterol, total proteins and albumin, total bilirubin, iron, glucose, uric acid, creatinine, and liver enzymes, such as transaminase alanine aminotransferase (ALT), aspartate aminotransferase (AST), gamma glutamyl transferase (gamma-GT), C-reactive protein (CRP), erythrocyte, white blood cell and platelet counts, hemoglobin concentration, mean cell volumes, mean cell hemoglobin concentrations, and insulin-like growth factor I (IGF-I) were evaluated at the time of recruitment.

The plasma FA profile and malondialdehyde (MDA), erythrocyte antioxidant enzymes and FA profile, were assessed at the start of the study and after 30 days of hospitalization during the 12-week controlled phase of the trial. The anthropometric, biochemical, nutritional, and dietary pattern methods used were previously described in detail [24].

### 2.2. Dietetic Program

All subjects were provided with three meals a day, which were prepared fresh each day in the hospital kitchen under the supervision of a dietitian. Diets were planned on a four-week rotating menu based on recommended caloric and macronutrient and micronutrient content [25]. A calibrated dietetic spring scale was used to weigh the items of food served to and returned by the subjects for three consecutive days at the beginning and end of the study. Nurses who served food to the subjects between meals recorded the quantity of food consumed in household measurements. The energy value and the macronutrient content of the consumed food was calculated by using a computer program (DR3 v3.1.0, Sintesi Informatica, Milan, Italy).

### 2.3. Physical Activity

All participants underwent a comprehensive, progressive physical fitness and muscle mass enhancement training program of moderate intensity [26]. The exercise intervention was supervised by trained personnel and included 20 minutes of exercise per day, five times per week, for 12 weeks. Each exercise session consisted of a 5-minute warm-up, 5 minutes of strengthening exercise, 5 minutes of balance and gait training, and 5 minutes of cool-down. The strengthening exercises were done in a progressive sequence from seated to standing positions [27]. For each type of exercise, participants were instructed to repeat the movements up to eight times. Intensity was maintained at approximately 12 to 14 on the Borg Rate of Perceived Exertion scale [28]. Each subject’s ability to increase intensity was assessed.

Compliance to the exercise program was set a priori at a minimum of 70%. The exercise sessions were done outdoors, weather permitting, and were performed as follows:

- Chair exercise: repetitions of toe raises, heel raises, knee lifts, knee extensions, and others were done while seated on a chair. Hip flexions, lateral leg raises, and repetitions of other exercises were done while standing upright behind the chair, holding the back of the chair for stability.
- Ankle-weight exercise: to strengthen the legs, a fixed weight was placed on the ankle while participants did strengthening exercises. Weights of 0.50, 0.75, 1.00, and 1.50 kg were prepared and used in accordance with each participant’s strength as the resistance progressively increased. The exercises using these ankle weights included seated knee flexion and extension as well as standing knee flexion and extensions.
- Exercises with a resistance band: resistance bands were used to strengthen the upper and lower body. Lower-body exercises included leg extension and hip flexion. Upper-body exercises included double-arm pull downs and bicep curls.
- Balance and gait training: exercises included standing on one leg, multidirectional weight shifts, tandem stand, and tandem walk. Participants practiced proper gait mechanics focused on maintaining stability during walking and increasing stride length, toe elevation of the forward limb, heel elevation of the rear limb, frequency of stepping, and a heel-floor angle. Exercises included raising the toes (dorsiflexion) during the forward swing of the leg, kicking off the floor with the ball of the foot, walking with directional changes, and gait pattern variations.

### 2.4. Dietary Supplementation

Participants were assigned to treatment or placebo groups, according to a coded block randomization table prepared by an independent statistician. The intervention treatment was a liquid supplement (32 g) containing essential amino acids (EAA), whey protein, and a vitamin D mixture (SAI nutrition Gardanne, France). The chemical composition and its effectiveness in counteracting sarcopenia were previously published (Supplemental Table S1). The control group was given a placebo consisting of an isocaloric amount of maltodextrin with the same flavor and appearance as the intervention product. The dietary supplement or placebo was administered orally with meals once a day at midday for 12 weeks.

Investigators were blinded to the randomization table, the code assignments, and the procedure. Enrolled patients were assigned a progressive number. The supplements were distributed to the patients daily by a research dietitian, who was blinded to the randomization schedule. All supplements were in powder form and packed in numerically coded bottles. Instructions on each bottle included the amount of water that needed to be added. Study participants mixed the water and content of the bottle, so the product was ready for consumption. Participants were instructed to consume their normal amounts of food together with the dietary supplement.

Safety was assessed by the absence of serious side effects from the supplement (e.g., gastrointestinal symptoms such as nausea and diarrhea). Each subject was interviewed daily by a dietician about any unwanted side effects. Compliance with consumption of the supplement and placebo was 100%. No participant refused to take the supplement, and no side effects were reported.

### 2.5. Blood Sampling

Fasting blood samples were collected, for routine biochemical and hemato-chemical analyses. For biochemical assays, each blood sample (3 mL) was drawn and collected in heparinized glass tubes. The heparin tubes after gentle agitation were centrifuged at 3000 rpm at 4 °C for 10 minutes. After centrifugation, aliquots of 300 μL of plasma were immediately separated and frozen in dry ice. Then the samples were stored at −80 °C until use. Erythrocytes or red blood cells (RBC) were washed in phosphate-buffered saline and aliquots of 150 μL were stored at −80 °C in isotonic buffer and butylated hydroxytoluene (0.2%) as an antioxidant until subsequent use for membrane purification.

### 2.6. Lipid Analysis

RBC membranes (Ghost) were prepared by lysis with hypotonic buffer (phosphate 5 mM, pH 8, EDTA 0.5 mM), precipitated by centrifugation and washed several times to eliminate hemoglobin residues. Ghosts were used to assess cell membrane fatty acids. To this aim, ghost lipids were extracted according to Folch [29] with three different chloroform/methanol mixtures, as previously described [30,31].

FA composition of plasma and RBC Ghost organic extract was determined by gas chromatography. FA methyl esters, obtained after derivatization with sodium methoxide in methanol 3.33% w/v, were injected into a gas chromatograph (Agilent Technologies 6850 Series II, Santa Clara, USA) equipped with a flame ionization detector and an Alltech capillary column AT-Silar (30 m × 0.32 mm i.e., film thickness of 0.25 µm) under the following experimental conditions: gas carrier: helium, temperature: injector 250 °C, detector 275 °C, oven 50 °C for 2 min, and rate of 10 °C min^−1^ until 200 °C for 20 min. A standard mixture containing FA methyl esters was injected for calibration. All samples were blinded for the treatment group (supplemented or control).

### 2.7. Plasma Malondialdehyde (MDA) Analysis and Erythrocyte Antioxidant Enzymes

Lipid peroxidation product levels, as MDA, were measured in plasma by high-performance liquid chromatography (Jasco, Tokyo, Japan) equipped with an auto-sampler and a UV detector as previously reported [32].

Erythrocyte lysates were obtained from washed cells after two cycles of freezing and unfreezing followed by centrifugation to discard cell membranes. The hemolyzed fraction was tested for catalase (CAT), glutathione peroxidase (GPX), glutathione reductase (GR), and superoxide dismutase (SOD) enzyme activities, as previously described [33]. Hemoglobin concentration was determined on RBC lysate and used to normalize enzyme determinations [34] and Ghost FA content.

To determine glutathione content (GSH+GSSG), fresh erythrocytes were treated with 10% metaphosphoric acid. The homogenate was centrifuged at 5000 g for 10 minutes at 4 °C, and the supernatant was assayed according to the method described by Griffith [35], with some slight modifications. The sulfhydryl group of GSH, also generated from glutathione GSSG, by adding GR, reacts with 5,5-dithio-bis-2-nitrobenzoic acid and produces a yellow colored 5-thio-2-nitrobenzoic acid (TNB). The rate of TNB production is directly proportional to this reaction, which, in turn, is directly proportional to the concentration of GSH in the sample. The measurement of the absorbance of TNB at 412 nm provides an accurate estimation of the GSH level present in the sample.

### 2.8. Statistical Analysis

The clinical study consisted of a 12-week, randomized, double-blind, placebo-controlled supplementation trial. Nevertheless, results reported in this paper were obtained after 30 days of treatment.

The correlation of clinical and metabolic characteristics with lipidomic and antioxidant status of all participants at recruitment was assessed with linear regression models. The Pearson *R* correlation coefficients and 95% confidence intervals (CIs) were computed.

Within and between-groups changes of fatty acids were assessed by means of regression models for repeated measures and calculation of Huber-White robust standard errors to account for intra-patient correlation of measures. Differences within/between groups and 95% confidence intervals were computed. These analyses should be regarded as exploratory. Model assumptions were assessed graphically. Stata 14 (StataCorp, College Station, TX, USA) was used for computation.

The calculation of the sample size was based on a previous study [24].

## 3. Results

One hundred sixty-two elders were enrolled in the clinical trial and 130 participants were randomized. Thirty-two individuals were excluded because they either declined to participate (*n* = 11) or had abnormal laboratory values (*n* = 21).

The baseline characteristics, body composition, and psychological scores for the supplemented (S) and placebo-treated (P) groups were similar during the study admission (Table 1). Dietary intakes during hospitalization were similar for both groups (Supplemental Table S2).

We previously reported the effects of 12 weeks of supplementation vs. placebo with physical training on body composition [24]. Changes in free fat mass (FFM) were significantly different between the two groups with a mean difference of 1.7 kg (95% CI 0.9–2.5, *p* < 0.001) and greater increases in the S group (1.4 kg, *p* < 0.001) and no significant change in the P group (0.3 kg).

In this paper, we try to depict the metabolic status of the frail sarcopenic elderly by analyzing different biochemical, metabolic and clinical parameters to find possible correlations that describe these patients.

In Table 2, the average baseline biochemical characteristics for all the elderly participants are reported. Searching for metabolic and biochemical markers of frailty in sarcopenic elderly patients, we analyzed the correlations of the different measured parameters at study enrolment.

**Figure 1:**
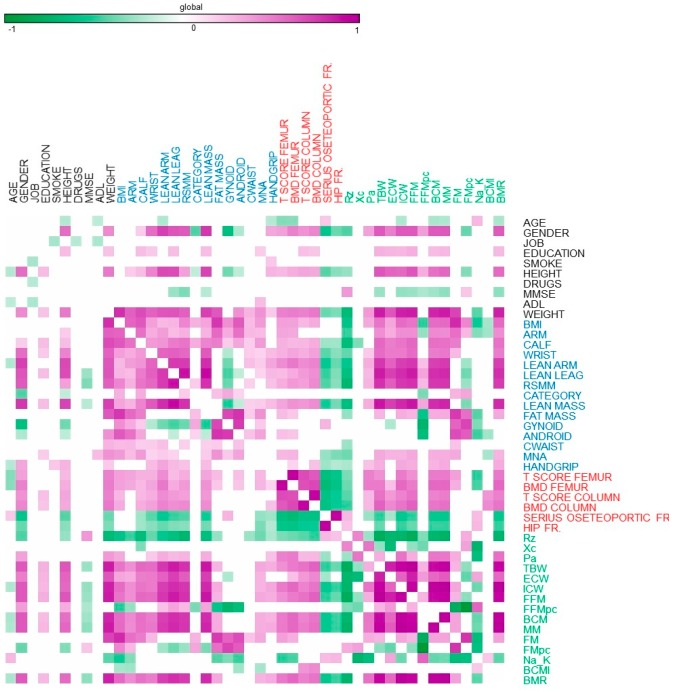
Heatmap reporting Pearson significant correlations (*p* < 0.05) among sarcopenic elderly features at recruitment.

Figure 1 depicts a heat map where we report the Pearson coefficients for statistically significant correlations (*p* < 0.05) between elderly features such as body composition, psychological scores, osteoporosis incidence, and bioimpedance indexes at the start of the study.

**Figure 2:**
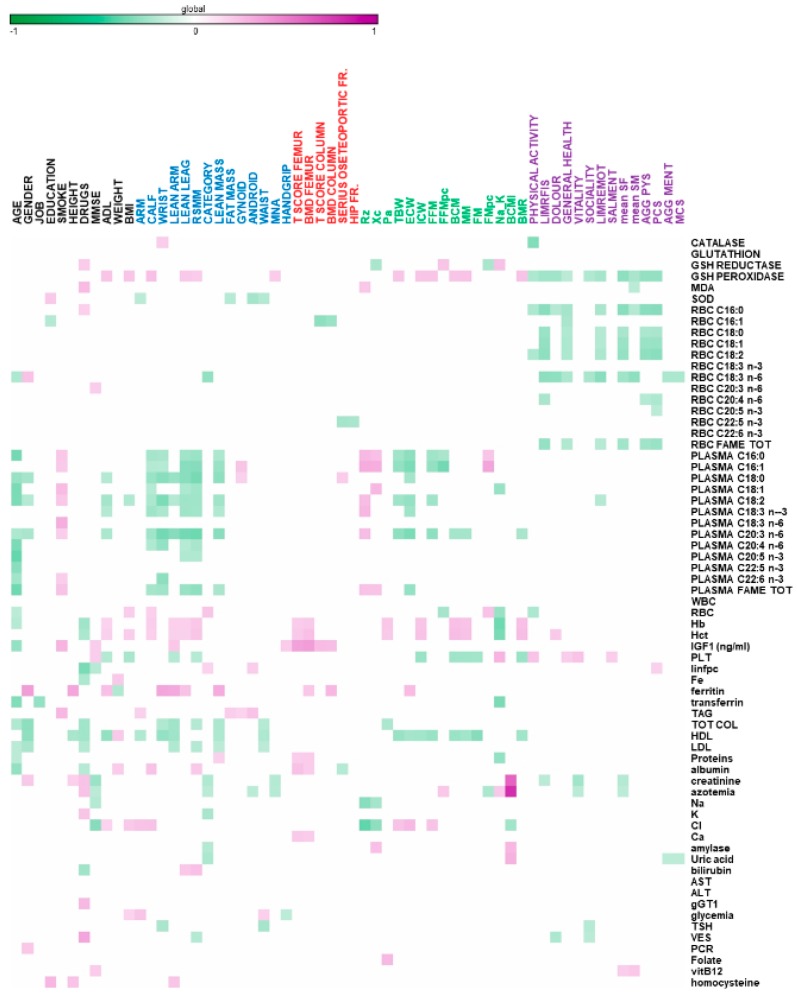
Heatmap reporting Pearson significant correlations (*p* < 0.05) among sarcopenic, elderly features versus hematological parameters, antioxidant status, and the lipidomic profile in plasma and red blood cells (RBC).

Furthermore, Figure 2 reports the correlations between biochemical parameters and patients’ characteristics. A negative correlation was observed between the plasma FA profile and age, and physical characteristics (underlined in blue). In particular, the saturated and omega-6 polyunsaturated fatty acids (PUFAs) display the highest correlation.

On the contrary, lipid composition of the ghost RBC showed a significant negative correlation with psychological scores (highlighted in violet), including palmitic and omega-6 PUFAs. The same correlations are displayed by RBC GPX activity.

**Figure 3:**
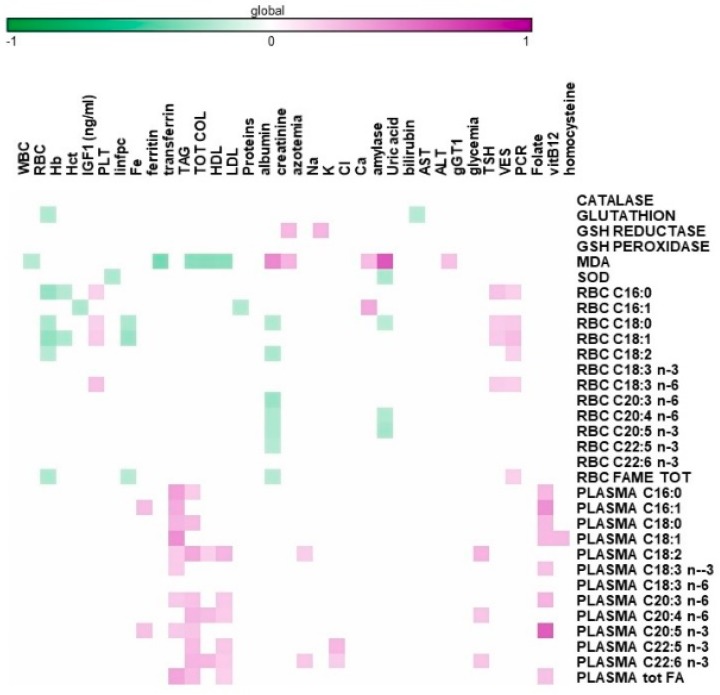
Heatmap reporting Pearson significant correlations (*p* < 0.05) among sarcopenic elderly hematological measures versus the antioxidant system and lipidomic profile in plasma and red blood cells (RBC).

Figure 3 reports significant correlations between the lipid profile and antioxidant potential measured in blood with the hemato-chemical parameters of the sarcopenic, elderly patients at the start of the study. It is worthy of note that the collected data demonstrate a positive correlation between specific plasma FAs and circulating neutral lipids such as LDL cholesterol and triglycerides. It is important to highlight the positive relationship between vitamin B12 and plasma FAs, which was more marked for eicosapentaenoic acid (EPA) in the sarcopenic, elderly patients.

RBC lipids, which include palmitic, stearic, and gamma-linolenic acids, positively correlate with inflammatory markers such as VES and PCR.

**Figure 4:**
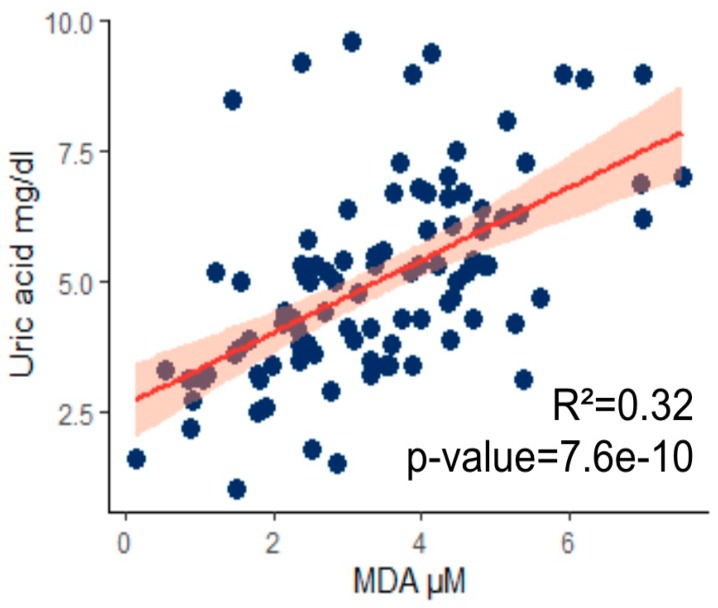
Uric acid in plasma of sarcopenic elderly correlates with malondialdehyde (MDA) content. Data correlated except for outlier values.

Regarding the antioxidant status, MDA levels negatively correlate with circulating lipoproteins while positively correlating with creatinemia, azotemia, and amylase. Of interest was the association between uric acid and MDA (*p* < 0.001) (Figure 4).

Moreover, we analyzed the short time effects, after 30 days, of the study protocol (dietetic regimen, exercise, and supplementation) on plasma lipid composition and antioxidant status. As shown in Table 3, the placebo group, during hospitalization, underwent combined physical activity with a standard diet program for one month, which shows a decrease of palmitic acid and saturated FAs (*p* = 0.007 and 0.012, respectively) with a significant increase of dihomo-gamma linolenic acid (DGLA) (*p* = 0.014). An increase in plasma MDA concentration and in the EPA proportion were also present, even if they were not significant (*p* = 0.084 and 0.076, respectively).

Supplementation combined with physical activity and a standard diet program induced a significant decrease of linoleic acid (LA) and omega-6 PUFAs (*p* = 0.002 and 0.028, respectively) together with an increase in plasma stearic and oleic acid concentrations (*p* = 0.001 and 0.046, respectively). Moreover, the antioxidant marker GR increased (*p* = 0.031, Table 3).

Table 4 shows changes over time between the groups. Significant differences were present for palmitic, stearic, and saturated FAs (*p* = 0.034, 0.011, and 0.008, respectively), which suggests that the supplement restores saturated FA levels that are consumed during physical activity.

## 4. Discussion

While the etiology of sarcopenia is multifactorial, a consensus is emerging that oxidative damage of skeletal muscle is a modifiable risk factor to consider in the development of this condition [36]. Physical activity, which can attenuate the development of sarcopenia, can also enhance production of ROS that adversely affects muscle membranes and promotes proteolysis of muscle [37].

The dietary intake of FAs is known to influence the FA composition of stored and structural lipids in different body compartments, such as erythrocytes and serum lipids [38,39,40,41]. Furthermore, dietary lipids impact the circulating FAs that, in turn, reflect the composition of the skeletal muscle membranes [18,42].

Alterations in membrane lipid composition play a central role in sarcopenia being able to modulate mitochondrial function. The FA membrane content may affect aging by protecting membranes against lipid peroxidation and preventing oxidative stress that influences membrane proteins and functions. The manipulation of membrane FA content by diets that differ in lipid composition induces changes in mitochondrial phospholipid FAs that reflect the PUFA profile of the dietary lipid sources [43]. The increased degree of saturation may protect against ROS in the skeletal muscle mitochondria, not only in relation to the decrease of lipid peroxides but also influencing mitochondrial respiration through a protonophoric effect on the inner cristae membrane, and an inhibitory action on the electron transfer chain.

The findings of the present study in the elderly provide evidence of a correlation among plasma and the RBC lipid phenotype with clinical, physical, and hematological characteristics. The possibility to ameliorate the lipid profile with diet, exercise, and supplementation is also presented.

Among the placebo-treated elders, consumption of a standard control-diet combined with regular exercise, even for a short time, induced a decrease of saturated FAs, especially palmitic acid, while the increase of MDA likely indicates the presence of oxidative stress induced by exercise.

Senescent muscle is susceptible to oxidative stress during exercise due to the age-related ultrastructural and biochemical changes that facilitate ROS formation. Furthermore, the reduction of muscle repair and regeneration capacity with old age could potentially enhance cellular oxidative damage. While endurance exercise is an effective way to maintain muscle mass during old age, it also increases free radical generation and, thus, the risk of oxidative damage to skeletal muscle [44].

On the contrary, the use of a dietary supplement, enriched with branched-chain amino acids (BCAAs), reverts the lipid phenotype of the patients. It appears that the fatty acid anabolism is stimulated by the supplement causing an increase of stearic acid and oleic acid, while palmitic acid levels, which decreased in patients in the placebo group, retain the same concentration. This suggests the possible conversion of BCAAs to lipids, or their utilization as an energy source in muscles during exercise. This preserves circulating saturated FAs.

Cross-sectional studies have suggested that elevations in plasma BCAAs are linked to markers of insulin resistance and adipose tissue FA synthesis. However, contrasting results are present in the literature [45]. For this reason, the correlation of BCAA use and the plasma lipidomic fingerprint is an important topic for future research because of the widespread use of BCAAs and whey protein as anabolic supplements. It is important to highlight that the decrease of LA and omega-6 PUFAs indicates a lower inflammatory state in the elderly subjects. Furthermore, supplemented subjects did not show an increase in MDA levels, but rather an induction of antioxidant enzymes, which may be related to the changes in muscle metabolism in the presence of BCAAs.

Fat is a major source of energy for resting and working muscles and may exert effects on age-related muscle loss through a number of mechanisms, including by influencing the FA composition of the sarcolemma membrane, increasing chronic low-grade inflammation (circulating cytokines), and diminishing insulin sensitivity [18,19,46,47].

The study by Sitnick et al. [48] sheds new light on the role of muscle and fat in lifestyle-related diseases. The researchers showed that chronic consumption of a high saturated fat and cholesterol-rich diet impairs the ability of murine skeletal muscle to hypertrophy in response to a mechanical load. Thus, these findings suggest that a high fat diet may not only increase fat mass but may also decrease muscle mass.

Nevertheless, to date, only two observational studies have correlated the FA profile with skeletal muscle mass [49,50]. These studies reported dietary fat intake among middle age and active people, which was estimated by food frequency questionnaires and not by blood FA profiling that reflects cell membrane composition. It is worth mentioning that these studies reported significant negative correlation coefficients between saturated fat intake and appendicular lean mass in adults (*r* = 0.97, *p* < 0.001, *r* = 0.99 in women, and *r* = 0.98 in men).

Low intensity exercise affects the blood FA profile. In the study of Andersson et al. [51] a group of sedentary middle-aged Swedish men, during 10 weeks, on a standardized diet with an identical fat composition, were randomized to remain sedentary or begin with low intensity physical activity. They demonstrated that exercise decreases skeletal muscle phospholipid FAs, especially palmitic acid, LA, and the sum of omega-6 PUFAs. Although a limitation of their study was the lack of a controlled diet, those results are consistent with the changes in the FA profile of the present study.

Moreover, recent findings indicate that long-term consumption of a high fat diet favors the accumulation of long-chain PUFAs in male rats, which induce the release of chemokines that may contribute to muscle mass loss [52].

Regarding the specific changes of the FA profile in our groups, an interesting and new finding of this study is the increase of oleic acid in association with the significant decrease in LA that was observed in the pre-supplemented and post-supplemented groups.

A previous animal model study demonstrated that oleic acid might play a favorable, pivotal role in skeletal muscle regeneration, while LA conversely impairs muscle regeneration [53].

Moreover, oleic acid improves the lipid profile [54], maintains a balance of body weight [55], and prevents palmitate-induced mitochondrial dysfunction, insulin resistance, and inflammatory signaling in neuronal cells [56] and skeletal muscle [57].

The increase of oleic acid may also explain the decrease of the CRP that was found in the present study, due to its anti-inflammatory activity [58] paralleled by the reduction of pro-inflammatory omega-6 PUFAs. Candow et al. [59] demonstrated that omega-6 PUFAs may augment the loss of muscle mass, and that the omega-6 to omega-3 PUFA ratio plays a key role in metabolic reactions in muscle tissues.

In the present study, the placebo group also showed beneficial lipid effects such as the decrease of palmitic acid and the significant increase of DGLA. It is well known that palmitic acid is positively associated with inflammation, such as a decrease of interleukin-6. In this regard, a reduction of palmitic acid may be a marker of inflammation reduction, even if, in our study, we did not show a correlation between saturated FAs and CRP [60].

In relation to the increase of DGLA (20:3 omega-6), it is produced by the metabolism of gamma-LA (18:3 omega-6) and can be incorporated into cellular glycerolipids (primarily phospholipids). Upon cell activation, membrane-bound DGLA is released by the action of enzyme phospholipase A2(s) and is converted into a mixture of metabolites with anti-inflammatory properties [61].

These lipidomic results sustain the hypothesis that a controlled diet with exercise may induce an anti-inflammatory response. Supplementation has propitiously affected the pro-antioxidant status. This is evident by the fact that GR levels increased significantly after supplementation whereas no changes were found among the placebo-treated subjects. Few data are present in the literature concerning whey protein supplementation and antioxidant metabolism, which are mainly related to GSH synthesis [62].

The molecular mechanisms involved in the development of sarcopenia are still not fully understood. However, there is a general consensus regarding the role of oxidative damage to muscle cells in sarcopenia and muscle aging [6,63].

Several biochemical processes are involved in aging-associated muscular atrophy. Sarcopenia mainly affects glycolytic fibers [64], which causes atrophy [65] via increased proteolytic activity [66,67]. It also makes them overproduce ROS [7,14,68]. Lastly, it destroys them through apoptosis or programmed cell death [69,70]. All these processes could result from abnormal mitochondrial function, since they are the main site of substrate oxidation, ROS production, and apoptosis.

These changes, which occur during aging, cannot be reversed but they may be reduced and controlled. Dietary supplementation might be beneficial in countering the effects of aging and age-related diseases. We speculate that the increase of the antioxidant enzyme GR, as observed in the treated group, was due to the daily intake of vitamin D contained in the dietary supplement. A recent study reported a novel link between vitamin D supplementation and improvement in cellular GSH. Using a monocyte cell model, the study by Ruggiero et al. demonstrated that vitamin D up-regulates the enzymes glutamate cysteine ligase (GCLC) and GR [71]. Then, some beneficial effects of vitamin D supplementation may be mediated by an improvement in the cellular reduced GSH levels and a decrease in ROS and pro-inflammatory cytokines.

One of the limitations of our study is the lack of serum vitamin D assessment of the patients. As such, we cannot consider whether there is a correlation between these parameters. Therefore, these hypotheses will need to be investigated in future clinical trials.

Serum uric acid (UA) in humans is the end product of purine metabolism, and a number of studies have shown that hyperuricemia is an important risk factor for systemic inflammation [72], endothelial dysfunction [73], hypertension [74], impaired fasting glucose [75], cardiovascular disease (CVD), and mortality [76]. Although a strong association exists among serum UA levels and various CVDs, a normal level of UA has, so far, not been proven to have a pathogenic role but, instead, is considered to be the ROS scavenger with strong antioxidant properties [77,78].

Thus, increased UA levels within the normal range may play an important protective role in counteracting the excessive production of free radicals, especially in elderly patients. We performed an exploratory analysis and found a correlation between UA and MDA (Figure 4) demonstrating that, even in sarcopenic subjects, there is a relationship between oxidative stress and the UA level.

Various studies demonstrated that a higher circulating level of serum UA is prospectively associated with higher handgrip strength in middle-aged and older individuals [79,80,81]. Our study has not highlighted this correlation with muscle function.

## 5. Conclusions

In conclusion, this is the first study in the literature that describes the plasma lipid profile together with pro-antioxidant status in a group of sarcopenic subjects before and after a randomized dietary supplementation trial with personalized aerobic exercise and endurance training.

In both groups, we found, after 4 weeks of treatment, a change in the framework of FAs. The evidence presented in this study suggests that dietary changes and physical activity are key factors involved in the amelioration of the FA profile. The treated group, however, has the advantage of a favorable increase in oleic acid associated with a decrease in LA and omega-6 PUFAs. In addition, only the treated group showed improvement in antioxidant markers, which demonstrated an increase in GR levels. 

The study of the lipid profile in association with the study of the balance pro-antioxidant represents a very interesting field of research in sarcopenic patients that has to be further explored and expanded in detail.

## Figures and Tables

**Table 1:** Baseline characteristics of the participants ^1^.

	Supplement Group (69 Subjects)	Placebo Group (61 Subjects)
Characteristics		
Age (y)	80.77 ± 6.29	80.21 ± 8.54
Male *N* (%)	29 (42%)	24 (39%)
Smokers *N* (%)	3 (4%)	5 (8%)
Level of schooling (years)	7 (3–11)	5 (2–9)
Body composition		
Fat free mass (kg)	39.90 ± 8.13	38.71 ± 8.37
Fat mass (kg)	17.81 ± 6.78	19.21 ± 9.18
Gynoid (%)	35.79 ± 9.67	37.67 ± 10.60
Android (%)	34.21 ± 10.76	34.26 ± 12.85
RSMM (kg/m^2^)	6.60 ± 1.19	6.36 ± 1.32
MNA (score)	17.84 ± 3.07	17.84 ± 3.57
Weight (kg)	59.47 ± 11.16	59.39 ± 13.51
BMI (kg/m^2^)	23.85 ± 3.63	23.93 ± 4.60
Wrist circumference (cm)	16.29 ± 1.75	16.08 ± 1.42
Arm circumference (cm)	25.22 ± 3.36	25.02 ± 3.80
Calf circumference (cm)	30.43 ± 3.13	29.95 ± 4.55
Waist circumference (cm)	88.95 ± 9.74	89.01 ± 10.15
Handgrip (kg)	16.63 ± 4.99	19.62 ± 6.01
Psychological scores		
MMSE (score)	21.78 ± 3.70	20.5 ± 4.93
ADL (score)	3.97 ± 1.19	4.03 ± 1.08
SF-36 MCS (score)	46.65 ± 10.7	44.0 ± 9.7
SF-36 PCS (score)	34.1 ± 10.2	37.1 ± 11.0

^1^ Data are shown as mean ± SD, median (25th–75th) unless otherwise specified [24]. RSMM = Relative Skeletal Muscle Mass. MNA = Mini Nutritional Assessment. BMI = Body Mass Index. MMSE = Mini Mental State Examination. ADL = Activity Daily Living. SF-36 MSC = SF-36 mental components. SF-36 PCS = SF-36 physical components.

**Table 2:** Baseline biochemical parameters of the participants ^1^.

Hematology	Supplemented Group	Placebo Group
Proteins (g/dL)	6.67 ± 0.55	6.56 ± 0.61
Albumin (g/dL)	3.76 ± 0.54	3.6 ± 0.55
Albumin (%)	55.92 ± 5.82	54.83 ± 6.00
Creatinine (mg/dL)	0.95 ± 0.7	0.91 ± 0.38
Azotaemia (mg/dL)	45.00 ± 35.41	46.92 ± 22.42
Uric Acid (mg/dL)	5.00 ± 2.20	5.15 ± 1.89
CRP (mg/L)	0.30 (0.14–1.23)	0.33 (0.16–1.03)
IGF-I (ng/mL)	80.6 ± 33.8	82.7 ± 38.8
White Blood Cell (10^9^/L)	6.96 ± 2.06	7.39 ± 3.08
Red Blood Cell (10^12^/L)	4.27 ± 0.48	4.24 ± 0.84
Hemoglobin (g/dL)	12.60 ± 1.47	12.19 ± 1.77
Hematocrit %	38.20 ± 4.25	36.91 ± 6.31
Platelet count (10^9^/L)	245.94 ± 85.38	240.14 ± 147.75
Lymphocyte count (10^9^/L)	1.80 ± 0.59	2.38 ± 3.88
Lymphocytes %	27.18 ± 8.51	25.92 ± 10.88
Iron (µg/dL)	72.46 ± 30.65	65.60 ± 30.79
Transferrin (g/L)	2.19 ± 0.50	2.15 ± 0.76
Glycaemia mmol/L	5.96 ± 2.12	5.51 ± 1.78
Triglyceride mmol/L	3.01 ± 1.09	3.32 ± 1.92
Total Cholesterol mmol/L	5.05 ± 1.21	4.9 ± 1.42
High-Density Lipoprotein mmol/L	1.35 ± 0.41	1.17 ± 0.38
Low-Density Lipoprotein mmol/L	3.14 ± 1.02	2.71 ± 1.48
Sodium (mEq/L)	138.88 ± 2.74	139.43 ± 2.92
Potassium (mEq/L)	4.06 ± 0.42	4.25 ± 0.61
Chlorine (mEq/L)	103.71 ± 3.86	104.83 ± 4.24
Calcium (mg/dL)	9.15 ± 0.63	9.02 ± 0.54
Amylase (U/L)	32.53 ± 24.31	28.60 ± 12.40
Total Bilirubin (mg/dL)	0.74 ± 0.47	0.71 ± 0.35
AST (U/L)	19.01 ± 10.54	21.52 ± 19.19
ALT (U/L)	16.35 ± 11.94	20.08 ± 18.22
gGT (U/L)	32.31 ± 38.43	36.70 ± 42.17

^1^ Data are shown as mean ± SD. Median (25th–75th) unless otherwise specified [24]. CRP = C-Reactive Protein. IGF-I = insulin like growth factor-I.

**Table 3:** Effects of a 30-day clinical trial on plasma fatty acids and oxidative stress markers of physical activity and amino acid supplementation in elderly sarcopenic subjects (*n* = 69 in the supplementation group, *n* = 61 in the placebo group).

Fatty Acid	Placebo Group			Supplemented Group		
	PRE	POST	*p* Value	PRE	POST	*p* Value
Palmitic acid (C16:0)	30.1 ± 3.52	28.1 ± 3.70	0.007	29.2 ± 3.01	29.3 ± 2.95	0.843
Palmitoleic acid (C16:1)	3.63 ± 1.14	3.83 ± 1.52	0.209	3.63 ± 1.25	3.55 ± 0.99	0.528
Stearic acid (C18:0)	5.75 ± 0.95	5.76 ± 1.02	0.952	5.61 ± 0.69	6.08 ± 0.85	0.001
Oleic acid (C18:1)	24.2 ± 6.44	25.4 ± 3.73	0.265	24.1 ± 3.21	25.0 ± 2.79	0.046
Linoleic acid (C18:2)	22.9 ± 4.19	22.1 ± 3.80	0.190	23.2 ± 3.23	21.6 ± 3.81	0.002
α-linolenic acid (C18:3 *n*-3)	0.44 ± 0.17	0.48 ± 0.18	0.206	0.53 ± 0.29	0.60 ± 0.52	0.347
γ-linolenic acid (C18:3 *n*-6)	0.23 ± 0.18	0.30 ± 0.29	0.191	0.25 ± 0.30	0.28 ± 0.25	0.504
Dihomo-γ-linolenic (C20:3 *n*-6)	1.30 ± 0.34	1.44 ± 0.39	0.014	1.36 ± 0.91	1.44 ± 0.32	0.496
Arachidonic acid (C20:4 *n*-6)	9.24 ± 2.48	10.31 ± 7.27	0.325	9.64 ± 2.73	9.81 ± 2.67	0.655
Eicosapentaenoic acid (C20:5 *n*-3)	0.34 ± 0.19	0.43 ± 0.17	0.076	0.42 ± 0.15	0.45 ± 0.18	0.199
Docosapentaenoic acid (C22:5 *n*-3)	0.42 ± 0.18	0.41 ± 0.10	0.593	0.42 ± 0.12	0.43 ± 0.10	0.694
Docosahexaenoic acid (C22:6 *n*-3)	1.50 ± 0.42	1.49 ± 0.41	0.927	1.57 ± 0.58	1.51 ± 0.48	0.424
AA/EPA	29.3 ± 12.3	26.3 ± 16.6	0.299	24.9 ± 8.56	24.5 ± 9.77	0.809
Saturated fatty acids	35.8 ± 3.75	33.9 ± 3.94	0.012	34.8 ± 2.99	35.4 ± 3.21	0.229
Monounsaturated fatty acids	27.8 ± 6.47	29.2 ± 4.21	0.168	27.8 ± 3.65	28.5 ± 3.02	0.112
Polyunsaturated fatty acids	36.4 ± 5.4	36.9 ± 6.21	0.604	37.6 ± 4.56	36.1 ± 4.27	0.024
Omega-6	33.6 ± 5.23	34.1 ± 6.21	0.654	34.5 ± 4.35	33.1 ± 4.13	0.028
Omega-3	2.73 ± 0.57	2.81 ± 0.54	0.381	2.93 ± 0.80	3.00 ± 0.83	0.618
Omega-6/Omega-3 ratio	12.9 ± 3.97	12.7 ± 4.32	0.809	12.4 ± 2.84	11.8 ± 3.23	0.155
Plasma Malondialdehyde (µM)	3.78 ± 1.91	4.20 ± 1.98	0.084	3.42 ± 1.68	3.51 ± 1.75	0.663
Superoxide dismutase (U/g Hb)	4724 ± 2499	4800 ± 2868	0.891	4413 ± 2520	4445 ± 2269	0.937
Total GSH (nmol/mg Hb)	4.08 ± 1.45	4.33 ± 1.93	0.408	4.35 ± 1.23	4.34 ± 1.61	0.981
Glutathione Reductase (U/g Hb)	3.06 ± 1.16	3.23 ± 1.13	0.352	2.59 ± 0.66	2.85 ± 0.88	0.031
Glutathione Peroxidase (U/g Hb)	28.6 ± 16.2	30.2 ± 16.0	0.553	36.3 ± 13.6	31.2 ± 18.1	0.084

Fatty acids are reported as the percentage of total plasma fatty acids. Malondialdehyde was assayed in plasma, while antioxidant enzymes and glutathione were assayed in erythrocyte lysates and normalized to hemoglobin content (Hb). AA/EPA = Arachidonic acid/Eicosapentaenoic acid.

**Table 4:** Between group differences on plasma fatty acids and oxidative stress markers of the sarcopenic elderly. The placebo group was under a controlled diet and performs physical activity, while the supplemented group also takes the dietary supplement (*n* = 69 in the supplementation group, *n* = 61 in the placebo group).

Variables	Δ (95%) Between Groups	*p* Value Treatment Effect
Palmitic acid	1.73 (0.13 to 3.33)	0.034
Palmitoleic acid	−0.31 (−0.71 to 0.10)	0.137
Stearic acid	0.58 (0.14 to 1.02)	0.011
Oleic acid	−0.69 (−2.80 to 1.42)	0.520
Linoleic acid	−0.25 (−1.88 to 1.39)	0.765
α-linolenic acid	0.02 (−0.17 to 0.22)	0.799
γ-linolenic acid	0.03 (−0.10 to 0.16)	0.656
Di-homo-γ-linolenic	−0.08 (−0.39 to 0.23)	0.622
Arachidonic acid	−0.96 (−3.05 to 1.14)	0.366
Eicosapentaenoic acid	−0.02 (−0.09 to 0.05)	0.622
Docosapentaenoic acid	−0.00 (−0.05 to 0.05)	0.912
Docosahexaenoic acid	-0.09 (-0.28 to 0.10)	0.349
AA/EPA	1.19 (−4.89 to 7.27)	0.699
Saturated fatty acids	2.31 (0.61 to 4.01)	0.008
Monounsaturated fatty acids	−0.99 (−3.11 to 1.12)	0.354
Polyunsaturated fatty acids	−1.47 (−3.87 to 0.93)	0.226
Omega-6	−1.25 (−3.66 to 1.15)	0.303
Omega-3	−0.07 (−0.40 to 0.26)	0.673
Omega-6/Omega-3	0.27 (−1.48 to 2.01)	0.763
Malondialdehyde	−0.36 (−0.98 to 0.25)	0.243
Superoxide dismutase	702 (−682 to 2086)	0.316
Glutathione	0.02 (−0.54 to 0.58)	0.951
Glutathione reductase	−0.002 (−0.41 to 0.41)	0.993
Glutathione peroxidase	−7.71 (−15.92 to 0.49)	0.065

Fatty acids and Malondialdehyde were assayed in plasma, while antioxidant enzymes and glutathione were assayed in erythrocyte lysates and were normalized to hemoglobin content (Hb). AA/EPA = Arachidonic acid/Eicosapentaenoic acid.

## Supplementary Materials

The following are available online at https://www.mdpi.com/2072-6643/11/11/2569/s1. Table S1: Nutritional composition of the dietary supplement. Table S2: Dietary intakes of supplemented (*n* = 69) and control patients (*n* = 61) at baseline and after 12 weeks.
