# Supplementary material for: Fatty Acid Profile and Antioxidant Status Fingerprint in Sarcopenic Elderly Patients: Role of Diet and Exercise
Source: Nutrients. 2019 Oct 24;11(11):2569. doi: 10.3390/nu11112569 (PMC6893529; doi:10.3390/nu11112569)
Supplement: Supplementary file 1 [file nutrients-11-02569-s001.pdf]

**SUPPLEMENTAL TABLE S1.** Nutritional composition of the dietary supplement ([24])

| Energy value | Per 100 g | Per 32 g Dose | % RDA[1] per Dose |
|---|---|---|---|
| KJ | 1466 | 469 | |
| Kcal | 351 | 112 | |
| **Nutrients** | | | |
| Proteins (g) | 68.9 | 22 | |
| Lipids (g) | 1.1 | 0.4 | |
| Saturated fatty acids (g) | 0.2 | 0.0 | |
| Total carbohydrates (g) | 14.8 | 4.7 | |
| Simple carbohydrates[2] (g) | 2.6 | 0.8 | |
| Complex carbohydrates[2] (g) | 3.9 | 1.2 | |
| Polyols (g) | 8.3 | 2.7 | |
| Fiber (g) | 6.9 | 2.2 | |
| Fructo-oligo-saccharide (FOS) (g) | 3.2 | 1.0 | |
| **Minerals** | | | |
| Calcium (mg) | 25.8 | 8.3 | 1% |
| Phosphorus (mg) | 76.3 | 24.4 | 3% |
| Sodium (mg) | 917.4 | 293.6 | |
| Magnesium (mg) | 140.7 | 45.0 | 12% |
| Iron (mg) | 0.8 | 0.3 | 2% |
| **Vitamins** | | | |
| D3 (Cholecalciferol) (µg=IU) | 7.8=312 | 2.5=100 | 50% |

[1]RDA, Recommended Dietary Allowance. [2]Sugars and polysaccharides,

**Supplemental TABLE S2.** Dietary intakes of supplemented (n=69) and control patients (n=61) at baseline and after 12 weeks.

| | Placebo group | | Supplemented group | | p value[1] | p value[2] |
|---|---|---|---|---|---|---|
| | baseline | 12 weeks | baseline | 12 weeks | | |
| Energy (Kcal/day) | 1622±350 | 1615±273 | 1600±215 | 1573±339 | ns | ns |
| Proteins (g/day) | 59±8 | 60±9 | 54±12 | 55±11 | ns | ns |
| Fat (g/day) | 54±12 | 55±11 | 52±9 | 53±14 | ns | ns |
| Carbohydrates (g/day) | 225±4 | 220±5 | 214±3 | 212±4 | ns | ns |
| Vitamin D (UI/day) | 299±79 | 298±87 | 301±92 | 296±89 | ns | ns |

Data are expressed as mean ± standard deviation (SD) and carried out by Carnovale E. Marletta L. “Food composition tables”, Italian National Institute of Nutrition, Rome 1997.

Regression model for repeated measures: [1]p-value from within treatment regression model – test for time; [2]p-value from between treatment regression model -test for interaction; ns: not significant
